# Participants’ characteristics and motivations to screen for HIV vaccine and monoclonal antibody trials in KwaZulu-Natal, South Africa

**DOI:** 10.1186/s13063-021-05792-7

**Published:** 2021-12-11

**Authors:** Jill Hanass-Hancock, Bradley Carpenter, Tarylee Reddy, Ayanda Nzuza, Zakir Gaffoor, Ameena Goga, Michele Andrasik

**Affiliations:** 1 South African Medical Research Council, Gender and Health Research Unit, Cape Town, South Africa; 2 University of KwaZulu Natal School of Health Science, Durban, South Africa; 3 South African Medical Research Council, Biostatistics Research Unit, Seattle, USA; 4 South African Medical Research Council, HIV Prevention Research Unit, Seattle, USA; 5 HIV Vaccine Trials Network, Vaccine and Infectious Disease Division, Fred Hutchinson Cancer Research Center, Seattle, USA

**Keywords:** HIV prevention, Clinical trials, HVTN, HIV vaccine, Research participation

## Abstract

**Background:**

HIV is one of the greatest public health challenges in South Africa. Potential HIV vaccines and antibodies are thought to be cost-effective biomedical HIV prevention methods and are currently under investigation in phase I, II, and III trials. Consequently, current and future clinical trials need to ensure sufficient recruitment and retention. To achieve this goal, clinical trial staff need to understand the socio-demographic and behavioural characteristics of people volunteering to screen for these trials and their reasons for volunteering.

**Methods:**

We conducted a secondary analysis of participant screening data across five vaccine and monoclonal antibody trials at four sites in KwaZulu-Natal, South Africa. Our study reviewed the demographic, behavioural, motivational, and health-related data from the case report forms and screening questionnaires. Descriptive statistics, chi-squared, and one-way ANOVA tests were used to analyse participants’ characteristics and motivation to participate in HIV vaccine and monoclonal antibody trials. Analyses were conducted using R version 3.5.2.

**Results:**

Screening data from 1934 participants, including 79.2% of women, were obtained across all five trials (1034 enrolled, 900 screened out/declined). Screened participants predominately self-identified as black, heterosexual, cisgender women or men, many with lower educational backgrounds (43.9% did not complete secondary/high school), and several self-reported HIV-risk behaviours among themselves and their partners. 10.8% of the screened participants were living with HIV. Avoiding HIV risk was the main motivation to participate in clinical trials, followed by altruistic reasons such as a desire to help the community or helping to find a vaccine.

**Discussion:**

The current recruitment approach of these trials attracts heterosexual participants who seek to reduce HIV risk and support their community. Hence, the data suggest the need for and potential acceptance of continued ongoing HIV prevention efforts. Current trials attract participants with lower educational levels, which may be driven by the site locations, current community mobilisation strategies and research site opening hours. The sites could consider more flexible working hours to accommodate working participants and find ways to connect participants to educational support and opportunities to upgrade education levels for the current clientele.

**Trial registration:**

HVTN 100: A Safety and Immune Response Study of 2 Experimental HIV Vaccines, NCT02404311. Registered on March 17, 2015.

HVTN 111: Safety and Immune Response to a Clade C DNA HIV Vaccine, NCT02997969. Registered on December 16, 2016.

HVTN 108: Evaluating the Safety and Immunogenicity of HIV Clade C DNA Vaccine and MF59- or AS01B-Adjuvanted Clade C Env Protein Vaccines in Various Combinations in Healthy, HIV-Uninfected Adults, NCT02915016. Registered on September 22, 2016.

HVTN 702: Pivotal Phase 2b/3 ALVAC/Bivalent gp120/MF59 HIV Vaccine Prevention Safety and Efficacy Study in South Africa, NCT02968849. Registered on November 1, 2016.

HVTN 703/HPTN 081: Evaluating the Safety and Efficacy of the VRC01 Antibody in Reducing Acquisition of HIV-1 Infection in Women, NCT02568215. Registered on October 1, 2015.

## Background

In South Africa, HIV is still one of the greatest public health challenges and significantly affects the social and economic outcomes of the country [1–3]. The country has one of the highest HIV prevalence rates in the world (HSRC 2018, HIV prevalence nationally—14% [4]; Stats SA 2017, HIV prevalence 15–49 years—21.2% [5]). In addition, the national HIV incidence among 15–49-year-olds is very high, ranging from 0.79% [4] to 1.13% [6]. It is also well known that new HIV infections disproportionately affect young women in South Africa [7]. Nationally, the incidence among women aged 20–34 years is 1.51%, and among “black women aged 20–34 years”, it is 1.59% [4]. HIV prevalence and incidence also vary via province in South Africa. KwaZulu-Natal (KZN), which has one of the highest HIV prevalence rates in South Africa (prevalence 27%), is also one of the provinces with the highest new HIV infections [4]. The HIV incidence in KZN ranges in observational studies from 6.3/100 PY (person-years) to 14.8/100 PY and in clinical trials averages around 6.74 per 100PY [8, 9].

Potential vaccines are considered one of the most cost-effective biomedical prevention methods. Numerous efforts are currently underway to develop an HIV vaccine [10–12]. Monoclonal antibodies (mAbs) are another biomedical HIV prevention tool rapidly advancing through clinical research in multiple early phase one and late phase two clinical trials [10–13]. The HIV Vaccine Trials Network (HVTN) is one of the US National Institutes of Allergies and Infectious Diseases (NIAID) funded networks testing different versions of vaccines and, in partnership with the NIAID-funded HIV Prevention Trials Network (HPTN), testing monoclonal antibodies using intravenous infusion in South Africa [14].

Efforts have also been made to address the specific HIV strains prevalent in South Africa and the surrounding sub-Saharan African region through the phase 2b/3 HVTN 702 trial, which unfortunately has shown to be non-efficious [15]. Further developments are underway to evaluate the combination of pre-exposure prophylaxis (PrEP) and vaccines (PrEPVac trial) [10–12, 16]. Continued research is needed to understand the best application and roll-out of effective HIV prevention methods in countries like South Africa [12]. To develop and roll out an effective HIV prevention method in the public health domain of sub-Saharan Africa, decades of research are still needed. Future clinical trials will require many participants who are willing to test new HIV prevention methods in the region. Understanding participants’ characteristics and motivation for participating in HIV prevention trials in this region will aid efforts to strategise and refine screening procedures. Thus, ensuring appropriate recruitment and retention in these future trials.

Early speculations about participants’ characteristics and motivations predicted that, in sub-Saharan Africa, characteristics of participants will differ from resource-rich settings and that this will impact motivations to participate in particular, for phase 2 and 3 trials which enrol groups with heightened vulnerability to HIV [17]. It was argued that populations at highest risk for HIV are distinctively different in the heterosexually driven epidemic in the region than in other parts of the world where commercial sex workers, intravenous drug users (IDUs), men who have sex with men (MSM), and transgender persons are at highest risk [17]. Smit et al. argued that key populations such as sex workers, IDUs, MSM, and transgender persons demonstrate a higher HIV risk awareness than the general heterosexual population, which may increase willingness to participate in trials among individuals in these key populations [17]. Smit et al. were also concerned that heterosexual people might have lower HIV risk awareness and, therefore, may be more challenging to recruit [17]. Hence, initial concerns about vaccine trials in sub-Saharan Africa were related to the challenges of recruiting and retaining participants, whether heterosexual people were willing to enrol, and whether trial participation would increase risky sexual behaviour [17]. Since then, clinical trials for HIV prevention have been implemented in many sub-Saharan African countries, enrolling thousands of participants, debunking these initial concerns.

Literature on participants’ motivation to participate in HIV vaccine and mAb trials are predominately available from resource-rich settings such as the USA. In the USA, motivations among key populations participating in trials are predominantly related to altruistic reasons, wanting to ‘help find a vaccine’, and, to a lesser extent, receiving information or financial gains [18]. Literature on HIV vaccine and mAb trials in sub-Saharan Africa reports predominately on trial-specific outcomes and procedures and seldom on the characteristics or motivations of participants in these trials [14, 18, 19]. In addition, information is published only on enrolled participants—i.e. people who test negative for HIV at screening and, at enrolment, are in good health, and willing to enrol in these trials.

One of the few sub-Saharan papers providing information on motivation to participate in HIV prevention trials provides data from enrolled police officers in Dar es Salaam, Tanzania [20]. The study revealed that these participants cited altruism as their primary motivation, followed by the desire to become a role model, the explanation of researchers, and the desire to know more about HIV prevention [20]. Protection against HIV, the magnitude of the disease in the community, and the impact of the disease on their own family were the least cited reasons. Interestingly, motivations differed based on sex, marital status, and level of education [20]. Male participants with higher education and unmarried participants were more likely to cite the researcher’s explanation as a motivation to participate. Participants with lower education levels were more likely to cite ‘knowing one’s health status’ as a motivation to participate [20].

Another study focusing on enrolled participants in vaccine trials in Nairobi, Kenya, revealed similar results showing that participants mainly cited collaboration with science, altruism, and health benefits. To a lesser degree, participants cited financial gains as a motivator [21]. Unlike Tanzania, the Kenyan study found no association between motivations and sex or education level. Financial gains, however, were more likely to be cited as motivation for younger participants [21]. Both studies did not report on participants who screened out or declined, nor did they consider how the participants’ behavioural HIV-risk profile might impact motivations. There is as yet no South African study revealing the characteristics and motivations of participants in vaccine and mAb trials.

Hence, little is known about people who enrol, decline participation, or screen out of clinical trials in South Africa. Gaining a better understanding of both enrolled and not enrolled participants (i.e. screened out/declined) and how this relates to motivation to participate in trials will improve recruitment, retention, development of appropriate support, and engagement with participants and their communities.

This paper aims to describe the demographic and behavioural characteristics and motivations of people participating in vaccine and mAb trials at four sites within one clinical trial unit (CTU) in KZN, South Africa. Over the last decade, these clinical trial sites have implemented several phase 1, 2, and 3 vaccine and mAb trials for the HVTN and HPTN. HIV prevalence and incidence is very high in the pool of participants from this CTU (incidence of 4–10/100 PY across the trials). Incidence rates are highest among women below the age of 25 years, those not with a stable partner, and those already diagnosed with a sexually transmitted infection (STI) [8, 22]. These sites generally work with a population at high risk of exposure to HIV, which could influence health-seeking behaviour and willingness to test new prevention methods.

In this paper, we accessed the routinely collected data for all vaccines and mAb trials conducted at this CTU to describe the participants’ demographic and behavioural characteristics and motivations to participate in these trials. This paper is the first in a series of articles exploring motivations to participate in trials and how these relate to demographic and behavioural characteristics.

## Methods

This paper presents a secondary analysis of existing cross-sectional data collected at screening, prior to enrolment, from participants across five vaccine and mAb trials in one clinical trial unit in KZN. Trials included three phase 1 trials - HVTN 100, 108, 111 and two efficacy trials - HVTN 702 and HVTN 703/HPTN 081. These trials include all vaccine and mAb trials conducted at this clinical trial unit across four sites between 2014 and 2018.

### Study sample

This study includes secondary data from both enrolled and not enrolled participants in all five trials in KwaZulu-Natal, which have extensively been described in previous work [22, 23]. Enrolled participants had to be healthy and aged 18 to 40 years. Pregnant women were excluded, and women were required to use contraceptives to prevent pregnancy during each trial. All enrolled participants were HIV seronegative at screening and enrolment. Participants in phase 1 trials were also required to be “assessed by the clinic staff as being at ‘low risk for HIV infection’ as per the HVTN low-risk guidelines for South Africa” [24, 25]. The phase 2b HVTN 703/HPTN 081 study and phase 2b/3 HVTN 702 study required that participants were at high HIV risk and “must have had sexual intercourse at least twice in the past 30 days” prior to screening. For the mAb trial, HVTN 703/HPTN 081, only women were eligible.

All screened participants were recruited from the greater eThekweni and surrounding areas, including rural, semi-rural, and urban settings. Each trial site engaged in community mobilisation and outreach activities prior to the start of the trial and informed potential participants about the opportunity to participate in these trials. Community engagement and mobilisation is predominately conducted during working hours (Monday to Friday, 8 am–4 pm). Potential participants were invited to receive further information at the trial sites where screening was conducted. Part of the screening process included the completion of a site-specific HIV risk assessment tool and standardised case report forms (CRFs). Thereafter, potential participants either declined to participate or completed screening and, if eligible, were offered to be enrolled in one of the trials. Hence, data were collected from individuals that declined to participate, were screened out, or were enrolled. For this paper, all screened individuals are considered ‘participants’ (enrolled and not enrolled).

### Routinely collected data in HVTN trials using CRFs

In all HVTN trials, data were collected via a set of standardised CRFs. Data collected at screening/enrolment include information on demographics, health, and behavioural data. The mAb trial did not collect behavioural data from participants that were not enrolled. As CRFs were standardised, it was possible to pool these studies and analyse the characteristics of participants across trials.

The demographic CRFs included age, race, sex, gender, sexual orientation, and education. The behavioural CRFs included data on individual sexual behaviour, partner sexual behaviour and drug and alcohol use. In these CRFs, individual sexual behaviour was assessed over the last three months, including number and type of partners, type of sex, frequency of sexual intercourse, condom use, transactional sex, and sex under the influence of alcohol or drugs. Condom use was assessed with a 3-point Likert scale (never, sometimes, or always). The participants use of drugs while having sex was assessed with a binominal scale (yes/no) and then a specifying section identifying the drug type. Practising transactional sex in the last three months was assessed with a dichotomous answer (yes/no). Participants were asked if they had been “given and/or received money, gifts, drugs, goods, shelter or services in exchange for vaginal or anal sex”. Similar questions with identical assessment periods were asked about participants’ partners.

In terms of high-risk sexual health behaviours, participants’ consumption of alcohol was assessed using a 5-point Likert scale (never, once a week, 2–3 times a week, 4–6 times a week, every day). Injecting drugs was assessed with a binominal scale (yes/no). Drug use of partners while having sex was assessed with binominal answers (yes, no/do not know).

**Fig. 1:**
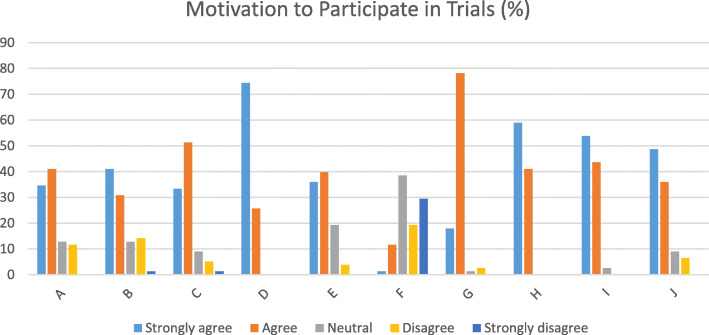
Motivations to participate in phase one trials. A = “I receive free counselling”; B = “I receive free HIV tests”; C = “I receive other tests or medical care for free or at no cost to me”; D = “I want to help find a vaccine that works for HIV prevention”; E = “The vaccine might protect me against HIV”; F = “I will be reimbursed or paid for being in this study”; G = “I will be informed by research” H = “It might help me to avoid high-risk behaviour”; I = “I am helping my community”; J = “I know someone who died of AIDS or who is HIV infected”

Screening also included HIV testing data as well as additional health data that were not analysed for this paper. Data on motivations were collected for phase one trials (selection of motivations listed in Fig. 1) but not for the efficacy trials. All trials collected data on the potential positive and negative social impacts of the study (e.g. positive: beneficial impact on personal relationships, feeling good helping others, improved medical care; negative: experience with friends or family, being turned away from a new job or educational programme, refusal of medical treatment).

The data were collected through the existing trials and underwent rigorous quality control. Data from enrolled participants were captured via Medidata or iDatafax and then uploaded on the Statistical Center for HIV/AIDS Research and Prevention (SCHARP) Atlas platform [26]. We retrieved the data from the SCHARP system after permission to access the data was granted [26].

### Additional data collection, entry, and quality control

In addition to the data from enrolled participants, we electronically captured all CRF data from participants who were not enrolled in these trials (i.e. individuals that declined to participate or screened out). This included the CRF screening data as well as reasons for screening out or declining (see Tables 1 and 2) that were collected and uploaded at the CTU but not on SCHARP (Statistical Center for HIV/AIDS Research Prevention). We also entered the CTU’s internal HIV risk screening questionnaire results for all participants and linked them via their Participant Identifier (PTID) into the dataset. This questionnaire provided additional behavioural data that we could use to identify HIV-risk profile groups. The questionnaire includes information on sexual behaviour, including the number and types of partners, sexual practices, condom use, transactional sex, and partner status. To ensure the accuracy of the self-entered data, we conducted a double-entry verification. In this process, 10% of the participants were randomly selected for a second independent research assistant to re-enter the data. Double-entered data were thereafter imported and merged in R, where validation of every entered field was conducted.

**Table 1:** Reasons for screening out

	Stratified by HVTN trials			
	100s^a^ 100/111/108	702^a^	703/HPTN081^b^	Sig.
***n***	85	410	166	
**HIV test result,** ***n*** **(%)**	15 (17.6)	152 (37.1)	42 (25.3)	NA
**HIV risk criteria,** ***n*** **(%)**	4 (4.7)	42 (10.2)	3 (1.8)	NA
**Pregnancy/breastfeeding, n(%)**	2 (2.4)	39 (9.5)	9 (5.4)	NA
**Unwilling to use effective contraception as defined in the protocol,** ***n*** **(%)**	4 (4.7)	5 (1.2)	2 (1.2)	NA
**Age,** ***n*** **(%)**	1 (1.2)	1 (0.2)	0 (0.0)	NA
**Physical exam findings, local lab results, and/or medical or psychiatric history or condition,** ***n*** **(%)**	57 (67.1)	139 (33.9)	105 (63.3)	NA
**Site assessment of participant’s availability,** ***n*** **(%)**	0 (0.0)	30 (7.3)	12 (7.2)	NA
**Ongoing or planned participation in another clinical trial**	1 (1.2)	0 (0.0)	1 (0.6)	NA
**Site assessment of participant’s understanding, n(%)**	1 (1.2)	2 (0.5)	0 (0.0)	NA
**Participant’s willingness to undergo HIV testing, counselling, and receive HIV test results,** ***n*** **(%)**	0 (0.0)	0 (0.0)	5 (3)	NA
**History of organ or tissue transplant,** ***n*** **(%)**	0 (0.0)	0 (0.0)	0 (0.0)	NA
**Receipt of experimental product, humanised/human mAbs, or contraindicated medications,** ***n*** **(%)**	0 (0.0)	0 (0.0)	0 (0.0)	NA

^a^Primary reason not enrolled

^b^Multiple reasons not enrolled

**Table 2:** Reasons for declining

	Stratified by HVTN trials			
	100s 100/111/108	702	703/HPTN081	Sig.
***n***	37	160	42	
**Reason not enrolled,** ***n*** **(%)**				NA
**Social pressure to not participate**	1 (2.7)	2 (1.2)	0 (0.0)	
**Unwilling to receive study product**	0 (0.0)	0 (0.0)	0 (0.0)	
**Individual changes his/her mind**	6 (16.2)	56 (35)	15 (35.7)	
**Unable to contact/no show**	28 (75.7)	58 (36.2)	11 (26.2)	
**Unable to comply with visit schedule**	2 (5.4)	28 (17.5)	8 (19)	
**Unwilling to risk testing positive on HIV test due to vaccine-induced seropositivity**	0 (0.0)	1 (0.6)	0 (0.0)	
**Unwilling to have IV infusion**	0 (0.0)	0 (0.0)	0 (0.0)	
**Other**	0 (0.0)	15 (9.4)	8 (19)	

### Determining behavioural HIV risk sub-groups

To synthesise the diverse set of behavioural questions from the behavioural CRFs, we developed an algorithm to simplify and summarise the questions into behavioural HIV risk profiles. This algorithm has been developed in alignment with UNAIDS standard terminology, where HIV risk is defined as “the risk of exposure to HIV or the likelihood that a person may acquire HIV” [27]. Using this terminology, behaviours rather than group membership (e.g. performing transactional sex but not belonging to the group of sex workers) are understood as placing individuals at risk of exposure to HIV. Behavioural characteristics such as individual risky sexual behaviour, intravenous drug use or sexual activity under the influence of drugs, exposure to sexual violence, transactional sex, and/or risky behaviour of sexual partners are seen as “creating, increasing or perpetuating risk” [27]. Based on this understanding, we have developed three behavioural HIV risk profiles and grouped participants fitting these HIV risk behaviours. We added another group of people who were already living with HIV at screening. Using this method, participants could fit into one or more of the four behavioural risk groups (algorithm is attached as a supplementary file). This algorithm allowed us to confirm the recruitment of individuals with low or high-risk profiles across the different trials and enabled us to sample purposely from this data set participants for further qualitative in-depth research.

Group1: Participants who reported individual behavioural risk factors such as:

- Sexual activity without consistent condom use (consistent = all the time) in the last three months with any of their partners
- Engagement in transactional sex
- Usage of intravenous drugs
- Been drunk or under the influence of drugs during sexual activities in the last 3 months

Group 2: Participants who reported any HIV risk behaviour among any of their partners

Group 3: Participants who reported *none* of the HIV risk behaviours above

Group 4: Participants who screened out of the study due to positive HIV status

### Statistical analysis

To simplify the analysis, we combined all phase 1 trial participants into one group (100s: HVTN 100, 108, and 111), used the South African HVTN 702 HIV vaccine trial as a second group (702) and the HVTN 703/HPTN 081 mAb trial as a third group (703/081) for the analysis.

The analysis focused on describing the population volunteering for HIV vaccine and mAb trials at the screening stage (enrolled, screened out participants and decliners) in terms of their socio-demographic characteristics and behavioural HIV risk factors (self-reported on behavioural risk assessment (BRA) and demographic (DEM) CRFs). In the first step, categorical variables from the CRFs (demographic, educational, HIV status at screening, and trial type/group) were summarised as frequencies and percentages, and continuous variables as means (with standard deviations) or medians (with interquartile ranges) where appropriate. From this, tables revealing participant characteristics in terms of their general demographics (sex, age group) as well as gender identification, sexual orientation and level of education were developed with their frequencies and percentages.

In the second step, the team developed an algorithm to define the different groups of participants with specific HIV risk profiles using the definition of behavioural risk provided above. The profiles were differentiated via type of risk (not degree/level of risk) and identified participants in terms of these profiles using the HVTN CRFs. Participants could fit into more than one risk profile group (except group 3).

Descriptive analyses were conducted separately for each trial. The chi-squared and one-way ANOVA tests, where appropriate, were used to assess whether participants’ characteristics differ between the trials. All analysis was conducted using R 3.5.2 with a *p* value of less than 0.05 considered statistically significant.

### Ethical considerations

The study accessed existing trial data. All trials have received ethical clearance prior to implementation. In addition, we received permission to use the data and funding from HVTN via the HVTN Initiative Programme (HIP). We also received ethical clearance for this study from the South African Medical Research Council (SMRC protocol ID number: EC003-2/2018). All trials, including the study presented here, were introduced and discussed with the community advisory committee at each trial site during their regular meetings.

## Results

### Participant demographic characteristics

Overall, demographic data from 1934 participants were obtained at screening across all five trials. Participants included 1034 enrolled participants and 900 participants who screened out before enrolment or declined to participate (Tables 3 and 4). 79.2% of the screened participants were women (female sex).

**Table 3:** Overview of the number of participants in each trial with available data

HVTN trial	Year collected	HVTN SCHARP data	Additional site data
100	2015	42 (enrolled)	48 (declined/screened out)
108	2017	3 (enrolled)	17 (declined/screened out)
111	2016	33 (enrolled)	57 (declined/screened out)
702	2017–2018	761 (enrolled) have full CRFs 281 of the above also have an additional risk assessment tool	570 (declined/screened out); have only demographic CRFs 228 of the above also have an additional risk assessment tool
703/HPTN 081	2016–2017	195 (enrolled)	42 with demographic data only (decliners) 166 (screened out)

**Table 4:** Full sample overview including all volunteers at screening (including those with demographic data only and those with demographic and behavioural data)

	Total	Stratified by HVTN study			
		100s 100/111/108	702	703/HPTN081	Sig.
***n***	1934	200	1331	403	
**Age (mean (SD))**	24.7 (4.5)	25.7 (5.5)	24.4 (4.2)	25.1 (5)	< 0.01
**Sex = female,** ***n*** **(%)**	1530 (79.2)	126 (63.3)	1001 (75.2)	403 (100)	< 0.01
**Education level,** ***n*** **(%)**					NA
**No formal education**	0 (0.0)	0 (0.0)	0 (0.0)	0 (0.0)	
**Some primary school**	8 (0.4)	1 (0.5)	6 (0.5)	1 (0.2)	
**Completed primary school**	38 (2)	7 (3.5)	17 (1.3)	14 (3.5)	
**Some secondary/high school**	801 (41.5)	78 (39.2)	532 (40.1)	191 (47.4)	
**Completed secondary/high school**	962 (49.8)	103 (51.8)	677 (51)	182 (45.2)	
**Some university/technical education**	96 (5)	3 (1.5)	81 (6.1)	12 (3)	
**Completed university/technical education**	15 (0.8)	7 (3.5)	7 (0.5)	1 (0.2)	
**National Certificate/Trade Certificate/*N***	7 (0.4)	0 (0.0)	6 (0.5)	1 (0.2)	
**Some graduate school**	1 (0.1)	0 (0.0)	1 (0.1)	0 (0.0)	
**Completed graduate school**	2 (0.1)	0 (0.0)	1 (0.1)	1 (0.2)	
**Prefer not to answer**	0 (0.0)	0 (0.0)	0 (0.0)	0 (0.0)	
**HIV+,** ***n*** **(%)**	209 (10.8)	15 (7.5)	152 (11.4)	42 (10.4)	0.24
**Enrolled,** ***n*** **(%)**	1034 (53.5)	78 (39)	761 (57.2)	195 (48.4)	< 0.01
**Screened out/declined,** ***n*** **(%)**	900 (46.5)	122 (61)	570 (42.8)	208 (51.6)	< 0.01
**Demographic data available,** ***n*** **(%)**	1934 (100)	200 (100)	1331 (100)	403 (100)	NA
**Behavioural data available,** ***n*** **(%)**	1156 (59.8)	200 (100)	761 (57.2)	195 (48.4)	< 0.01
**702 risk assessment tool data available,** ***n*** **(%)**	509 (26.3)	0 (0.0)	509 (38.2)	0 (0.0)	< 0.01
**All demographic and behavioural data available,** ***n*** **(%)**	676 (35)	200 (100)	281 (21.1)	195 (48.4)	< 0.01
**Some demographic and behavioural data available,** ***n*** **(%)**	1384 (71.6)	200 (100)	989 (74.3)	195 (48.4)	< 0.01
**Motivation for enrolment data available,** ***n*** **(%)**	78 (4)	78 (39)	0 (0.0)	0 (0.0)	< 0.01

Screened participants for the five trials were on average 24.7 years old, ranging from 18 to 44 (Table 4). While 56% of the participants had completed secondary/high school, a large percentage of participants (43.9%) had completed less than a secondary/high school education, and only a minority had enrolled in or completed any tertiary education or training (6.4%). In addition, 10.8% of participants screened for these trials were already living with HIV (tested positive at screening). The HIV prevalence data vary across trials, with an average of 7.5% in the phase 1 trials (100s), 11.4% in the 702 trial, and 10.4% in the 703/081 trial. Overall, the screening to enrolment ratio across all trials was 1.89, with enrolment ratios of 2.5, 1.75, and 2.07 in phase 1 (100s), 703/081, and 702 trials, respectively.

Complete behavioural and demographic data were available for 1384 participants, as some trials did not collect these data from individuals who declined to participate or screened out (Table 5). This smaller dataset included 65.5% of women (female sex), largely driven by the recruitment targets for some trials. Furthermore, 98% of participants were black and most identified as heterosexual (97.8%). Only 39 (2.2%) participants identified as ‘homosexual’, ‘gay’, or ‘other’ across all three trial phases. Except for one participant, who identified as other, all participants identified as either cisgender male or female.

**Table 5:** Demographics of sample with complete CRF data including basic behavioural and demographic data

	Stratified by HVTN trials			
	100s 100/111/108	702	703/HPTN 081	Sig.
***n***	200	989	195	
**Race,** ***n*** **(%)**				NA
**Black African**	197 (98.5)	985 (99.6)	185 (94.9)	
**White**	0 (0.0)	2 (0.2)	0 (0.0)	
**Indian**	2 (1)	1 (0.1)	9 (4.6)	
**Asian**	0 (0.0)	0 (0.0)	0 (0.0)	
**Mixed**	1 (0.5)	0 (0.0)	1 (0.5)	
**Other**	0 (0.0)	0 (0.0)	0 (0.0)	
**Sex at birth = female,** ***n*** **(%)**	127 (63.5)	717 (72.5)	195 (100)	< 0.01
**Gender identity,** ***n*** **(%)**				< 0.01
**Women**	127 (63.5)	717 (72.5)	195 (100)	
**Men**	72 (36)	272 (27.5)	0 (0.0)	
**Other**	1 (0.5)	0 (0.0)	0 (0.0)	
**Sexual orientation,** ***n*** **(%)**				NA
**Homosexual**	6 (3)	12 (1.2)	0 (0.0)	
**Heterosexual**	193 (97)	967 (97.8)	194 (99.5)	
**Bisexual**	0 (0.0)	9 (0.9)	0 (0.0)	
**Asexual**	0 (0.0)	0 (0.0)	0 (0.0)	
**Additional category**	0 (0.0)	0 (0.0)	0 (0.0)	
**Not sure**	0 (0.0)	1 (0.1)	0 (0.0)	
**Prefer not to answer**	0 (0.0)	0 (0.0)	1 (0.5)	

### Behavioural data

Not all trials include behavioural data collection for individuals that decline to participate or screened out; hence, data from some of these individuals were not collected via behavioural CRFs. For instance, the mAb study collects behavioural data from enrolled participants only. As a result, demographic and behavioural data were available for 1384 screened participants (1034 of those enrolled, Table 4). Using our studies’ risk-group algorithm, some participants fit into more than one behavioural HIV risk group. If a participant revealed that they did not use condoms consistently, they may also indicate that their partner has more than one sexual partner or does use drugs regularly (they would then fall into groups 1 and 2). Overall, 81.5% of screened participants indicated that they did not use a condom consistently. As expected, phase 1 trials had the lowest number of participants indicating a lack of condom use (22%). Conversely, a large percentage of participants in 702 (96.2%) and 703/081 (68.2%) reported not using condoms consistently.

Several participants also indicated at screening that they were using drugs or alcohol while having sex. Overall, this included 22% of the screened participants across all trials, with 2.5% in the 100s trials, 26.8% in 702 and 17.9% in the 703/081 trial. Similarly, 7.3% of the participants revealed that they practise transactional sex. This included only participants from 702 (8.7%) and 703/081 (7.7%). Not surprisingly, phase 1 trials had the most participants indicating that they were not exposed to behavioural HIV risk characteristics (77.5%). However, this also means that 22.5% of participants in trials that explicitly exclude individuals with higher HIV risk profiles did practise or were exposed to HIV risk factors. By comparison, less than 3% of participants, 2.8% in 702 and 2% in 703/081, reported low-risk profiles. The 703/081 trial did not include behavioural data on screened out individuals or decliners. Hence, Table 5 contains data on enrolled participants only for this study.

### Motivations to participate

To better understand the motivations to enrol or decline participation in trials, we analysed the information in the CRFs relating to ‘motivations’, reasons for screening out, and the local site records identifying reasons for declining.

Of the 661 screened out participants, 209 (32%) were ineligible because they were already living with HIV and 301 (46%) for other health reasons. Other reasons for being ineligible included: pregnancy or breastfeeding at the time of screening, being unwilling to use contraceptives, being unavailable for future visits, or not fulfilling the specific HIV risk criteria for a trial. Very few participants were ineligible for other reasons (Table 6).

**Table 6:** HIV risk groups of sample with at least basic behavioural and demographic data

	Total	Stratified by study			
		100s 100/111/108	702	703/HPTN 081	Sig.
***n***	1384	200	989	195	
**Group 1: individual risk**					
**Personal sexual risk**	1124 (81.5)	44 (22)	947 (96.2)	133 (68.2)	< 0.01
**Behaviour, yes** ***n*** **(%)**					
**Transactional sex, yes** ***n*** **(%)**	101 (7.3)	0 (0)	86 (8.7)	15 (7.7)	< 0.01
**Usage of drugs, yes** ***n*** **(%)**	305 (22)	5 (2.5)	265 (26.8)	35 (17.9)	< 0.01
**Group 2: Partner risk behaviour**					
**Indicated, yes** ***n*** **(%)**	528 (38.2)	6 (3)	459 (46.4)	63 (32.3)	< 0.01
**Group 3: No risks reported, n(%)**	222 (16)	155 (77.5)	28 (2.8)	39 (20)	< 0.01
**Group 4: HIV+, yes** ***n*** **(%)**	87 (6.3)	15 (7.5)	72 (7.3)	0 (0)	< 0.01
**Decliner, yes** ***n*** **(%)**	263 (19)	107 (53.5)	156 (15.8)	0 (0)	< 0.01

Although CRF data for the 239 individuals that declined to participate were limited, the most common reasons for declining were related to an inability to contact participants (40.6%) and participants ‘changing their mind’ (32.2%). Some participants (15.9%) could not comply with the visit schedule, and a few participants (1.3%) identified social pressure. No participants reported declining because they were uncomfortable with the intravenous infusion or study product injection (Table 1). More detail was not available from the CRF data.

We also assessed motivations to participate in phase 1 trials, as these data were unavailable for the efficacy trials. Screened participants agreed with most provided reasons (Fig. 1) but disagreed with reimbursement being a motivator. A desire to reduce HIV-risk behaviour (100%), develop a vaccine (100%), help the community (97.4%), informed of research (96.2%), free other tests and medicine (84.6%), and the fact that participants knew a person living with HIV (84.6%) were the most common reasons to participate in these trials. Free HIV testing (71.8%), free counselling (75.6%), the potential of the vaccine to protect from HIV (75.6%) and reimbursement (12.8%) were other reasons chosen to participate in trials.

## Discussion

Despite initial concerns, over the last decade, several vaccine and mAb trials have been implemented successfully in sub-Saharan Africa. This is, to our knowledge, the first time a dataset of this size has been used to assess participant characteristics and motivations.

Our data show that most of the participants in our trials identify as heterosexual and that they predominately report risky individual or partner sexual behaviour, and to a lesser degree transactional sex, as HIV risk behaviour. No participants reported intravenous drug use. The trials also included many young women (average age 25 years), but only a few people identified as ‘homosexual’, which is related to the trial’s eligibility criteria. Therefore, this study demonstrates that the recruitment of high-risk heterosexual groups for large phase 2 and 3 vaccine and mAb trials is possible in South Africa. Successfully enrolling participants with low-risk profiles in phase 1 trials is more challenging (see Table 5 risk groups).

The data also show that the trial sites are not recruiting other at-risk populations such as sex workers, transgender people, or men who have sex with men, even if the eligibility criteria allow their inclusion. The CRF data limit our ability to determine whether underrepresentation is related to the recruitment approach at these sites or whether certain at-risk groups are less likely to volunteer for these trials. Recruitment protocols need to become more inclusive of all at-risk groups and collect more detailed demographic data, particularly of underrepresented and marginalised groups. The 2021 UNAIDS ethics guidelines for HIV prevention trials, released after this paper was drafted, emphasise the need for fair and inclusive selection of populations [28]. The UNAIDS guidelines go much further and emphasise the need for inclusion of populations for which the current CRFs do not even collect demographic information (such as disability status) [28] and for which screening and trial procedures in their current form are inaccessible (e.g. absence of sign language, Braille, ramp, and disability accessible toilets at study sites).

Our study shows an overall enrolment of 53% across trials. This means that, even with intensive community engagement and mobilisation, clinical trials sites in this area of the world have a screening to enrolment ratio of approximately two screened participants for each enrolled participant. In addition, 10.8% of participants were determined to be living with HIV at screening. Considering that the community mobilisation and information emphasised that the trials could only enrol people with HIV-negative status, we assume that the 10.8% of participants living with HIV discovered their positive status at screening (proxy for ‘new HIV infections’). This information demonstrates the importance for trial sites to continue providing appropriate linkages to care for participants who have been screened and diagnosed with HIV infections and other health conditions. The data from the behavioural characteristics indicate several HIV risk behaviours among participants and their partners.

Expectedly, reducing HIV risk is one of the most endorsed motivations to participate in the trials at the four sites. The high HIV incidence rate and risk-behaviour data reinforce that these clinical trials work with participants at high risk of HIV infection, who may have more risk awareness, contributing to the participants’ motivations to enrol in clinical trials. The current management at these clinical trial sites attempts to reduce HIV risk among participants by providing health information, risk reduction counselling, and other prevention methods (PrEP and condoms). Our data demonstrate the need for this to be a continued focus. The CTU governing these sites also has a long-established memorandum of understanding with local public healthcare providers, which allows for the referral of participants to care and a provider of their choice, which are essential linkages that require continued facilitation.

Our demographic and behavioural data suggest that the sampled population comes from a disadvantaged background and is exposed to high HIV risks through situations and contexts that impact individual sexual behaviour. Participants are on average 25 years old, yet only a few participants have enrolled in or completed a post-school tertiary education or training. In fact, 43.9% of the participants have not completed school, which in South Africa is completed with matric at grade 12. Hence, education levels are much lower at these sites than in the Tanzanian and Kenyan studies, where 70% and 79% completed secondary or above education [20, 21]. Unfortunately, economic data are not available across these five trials. However, in South Africa, low educational attainment is associated with high unemployment and poverty [29]. Unemployed participants out of school are likely to be available for community engagement during the day on weekdays. Therefore, it is likely that the community engagement approach (weekday working hours) is more successful in recruiting the unemployed and those available during the day. Hence, education levels might be indirectly linked to trial participation. It might also be linked to the type of motivations reported in the CRFs. For instance, the Tanzanian study suggested that lower educational status is associated with participants engaging in vaccine trials to gain a better understanding of their health status. The sites in KZN provide access to free sexual reproductive health services and counselling and, therefore, may specifically attract individuals in need of these services. An in-depth analysis of this association between education and motivations could not be conducted with the CRF data as the sample of people with higher educational levels was too small.

Nevertheless, the data speak for the continued need to facilitate access to educational support by offering direct educational opportunities. The UNAIDS Ethical Guidelines on Clinical Trials emphasises that “people who could significantly benefit from new, safe and effective HIV prevention interventions often live in social or political contexts of vulnerability to exploitation, prosecution or other harms” [28]. Hence, HIV prevention trials need to reduce harm while maximising benefit without undue inducement. Increasing access to information and education could be one such benefit [28]. Although providing educational opportunities may be perceived as an undue inducement, not doing so may be viewed as a lack of social justice. More work is needed to establish the right balance between offering participation in research without undue inducement, increasing social justice, and supporting participants enrolled in research studies.

Considering that these trial sites approach disadvantaged populations, on-site researchers might find a review of the recruitment and community engagement strategies beneficial. Such a review should consider how other groups (e.g. people who are working, people of other sexual orientation and gender, people with disabilities) can be better accommodated to participate and how participants with low educational levels can be supported to understand clinical trials, its processes, and HIV-prevention options (e.g. continuing practices of simplifying informational material, use of infographics). In addition, better linkages to educational and economic opportunities should be explored. The Community care study at the Thembisa site in South Africa has begun to develop an approach that determines available community services and ways to improve their connection to the HIV care continuum [30]. Similar approaches should be tested in the KZN CTU to increase motivation to participate, improve social justice, and overall community engagement, recruitment, and retention in these trials.

For phase one trials, the data provide direct information on the motivations of participants. Motivations included a combination of wishing to reduce HIV risk, altruistic intentions (e.g. helping the community to develop a vaccine); personal benefits from participating in the trial (e.g. free counselling and tests; vaccine might protect them), as well as educational interest (e.g. being informed by research). Knowing someone living with HIV also contributed to most participants’ motivation, while reimbursement was not considered a motivator by most participants. Similar findings are made in Smit et al.’s review on sub-Saharan studies, Colfax in the USA and Andrasik et al. in the USA and Peru, who list altruism, desire to fight AIDS, but also material benefits as essential factors that influence motivations to participate in HIV vaccine trials [17, 18, 31]. Similarly, the Kenyan and Tanzanian study identify collaboration in research, altruism and, to some extent, knowledge about vaccine trials as major motivating factors driving participation in HIV vaccine phase 1 and 2 trials [20]. The main differences in the motivations of this CTU’s participants are related to the participants’ knowing somebody living with HIV and wanting to reduce their own HIV risk. Hence, the awareness of the general high HIV risk in the KZN population, the participants (and partners) own risk behaviour, and the fact that participants knew people living with HIV are motivating factors across these five trials.

Our work had several limitations: The analysis of screened participants is limited to the available data collected at screening for these trials. First, data on motivations was limited to phase 1 trials. Future efforts should include data from phase 2 and 3 trials. Second, for all vaccine and mAb trial participants, the CRF data provide limited information regarding socio-economic characteristics and motivations to participate or decline participation. This is related to limited data collection on screened out participants and decliners in general and inconsistent inclusion of behavioural and motivational CRFs in phase 2 and 3 trials. Including economic data will provide a more nuanced understanding of who participates in these trials. Third, the provided CRF’s reasons for declining are very generic (e.g. participant changed their mind) and provide little detail for analysis. Hence, future trials need to collect better data at screening.

More in-depth qualitative research must also be undertaken. Qualitative research needs to prompt experiences and perceptions from participants in different HIV risk groups and those enrolled, screened out, and declining enrolment. Furthermore, the cross-sectional analysis of screening data does not explain how sexual behaviour may change over time and whether this is associated with trial participation. Longitudinal and in-depth qualitative research is needed to understand participation in HIV vaccine trials better.

## Trial status

The HVTN 100 (NCT02404311) and HVTN 111 (NCT02997969) trials have been completed. The HVTN 108 (NCT02915016), HVTN 702 (NCT02968849), and HVTN 703/HPTN 081 (NCT02568215) trials are ongoing.

This work was supported by the NIAID US Public Health Service Grant UM1 AI068614 (LOC: HIV Vaccine Trials Network [HVTN]) as part of the HVTN Initiatives Program (HIP) and received ethical clearance from the South African Medical Research Council (protocol ID number: EC003-2/2018). The study has been completed.

## Data Availability

The study is accessing data through the HVTN network. Permission to access data will have to be requested from HVTN and SCHARP.

## Abbreviations

CRF: Case report form
HIV: Human immunodeficiency virus
HVTN: HIV Vaccine Trials Network
HPTN: HIV Prevention Trials Network
MTN: Microbicide Trials Network
PTID: Participant Identifier
SCHARP: Statistical Center for HIV/AIDS Research and Prevention
